# Red Palm Oil: Nutritional Composition, Bioactive Properties, and Potential Applications in Health and Cosmetics: A Narrative Review

**DOI:** 10.3390/molecules30224402

**Published:** 2025-11-14

**Authors:** Husna Madoromae, Monthon Lertcanawanichakul

**Affiliations:** School of Allied Health Sciences, Walailak University, Nakhon Si Thammarat 80160, Thailand; husna.md@mail.wu.ac.th

**Keywords:** red palm oil, bioactive compounds, antioxidant, anti-inflammatory, skin health, natural products, narrative review

## Abstract

Red palm oil (RPO) is a rich source of bioactive compounds, including carotenoids, tocopherols, tocotrienols, and polyphenols, which contribute to its potent antioxidant and anti-inflammatory properties. This review summarizes the current understanding of RPO composition, bioactivity, and potential applications in health and cosmetics. Current preclinical and small-scale clinical studies suggest that RPO bioactives can mitigate oxidative stress, modulate inflammatory pathways, and improve skin barrier function. Strategies to enhance stability and bioavailability, such as microencapsulation and formulation into emulsions or liposomes, are also discussed. The manuscript highlights the potential of RPO as a natural functional ingredient in dietary, nutraceutical, and cosmetic products. Comprehensive evaluation of these bioactive compounds provides insights for future research and practical applications in promoting human health.

## 1. Introduction

Red palm oil (RPO), obtained from the fruit of *Elaeis guineensis*, has garnered increasing scientific and industrial interest due to its unique chemical composition and multifunctional bioactivities. Unlike fully refined palm oil, minimally processed RPO retains high levels of carotenoids, including alpha-carotene, beta-carotene, and lycopene, as well as tocopherols, tocotrienols, polyphenols, and minor components such as phytosterols and squalene [1,2,3,4]. These bioactive constituents are sensitive to heat, light, and chemical processing, explaining the significant differences in composition between crude RPO and refined palm oil [2,5].

The carotenoids present in RPO serve as precursors for vitamin A, contributing to visual health and immune function, and act as potent antioxidants, scavenging reactive oxygen species (ROS) and reducing lipid peroxidation [3,6]. Tocotrienols, a form of vitamin E abundant in RPO, have been associated with neuroprotective, cardioprotective, and cholesterol-lowering effects, often exceeding the efficacy of tocopherols at similar concentrations [7,8]. Polyphenols and minor compounds such as squalene and phytosterols provide additional antioxidant, anti-inflammatory, and lipid-modulating effects, enhancing the overall bioactivity of RPO [9,10,11].

Beyond antioxidant activity, RPO exhibits antimicrobial properties against a range of Gram-positive and Gram-negative bacteria, including *Staphylococcus aureus*, *Listeria monocytogenes*, and *Escherichia coli*, suggesting potential applications in food preservation, oral care, and topical formulations [12,13,14,15]. Several in vitro and in vivo studies indicate that these effects result from synergistic interactions among carotenoids, tocotrienols, and phenolic compounds, which can disrupt bacterial cell membranes, inhibit biofilm formation, and modulate inflammatory pathways [16,17,18].

Emerging evidence also highlights RPO’s protective role against oxidative stress-related disorders, including cardiovascular disease, neurodegeneration, and skin aging [19,20,21]. Animal and human studies demonstrate that dietary supplementation with RPO can improve lipid profiles, enhance cognitive function, and mitigate UV-induced skin damage, supporting its multifunctional applications in nutrition and cosmetology [22,23,24].

This narrative review distinguishes itself from previous studies, which mainly focused on either the nutritional or bioactive components of RPO, by providing an integrated perspective on both health and cosmetic applications. It emphasizes mechanistic insights into antioxidant and anti-inflammatory pathways, explores strategies to enhance stability and bioavailability, and consolidates research from the past decade. By linking fundamental science, clinical evidence, and practical applications, this review guides the development of functional foods, nutraceuticals, and cosmeceuticals, offering a comprehensive framework for future research and product development.

## 2. Nutritional Composition of RPO

RPO is distinguished by its high content of fat-soluble vitamins and carotenoids. The main carotenoids present are alpha-carotene, beta-carotene, and lycopene, which act as vitamin A precursors and potent antioxidants [1,2,3,4] (Table 1). Total carotenoid content in crude RPO typically ranges from 500 to 700 mg/kg depending on fruit ripeness, extraction method, and geographic origin [3,4]. Tocotrienols, a subclass of vitamin E, are present at levels ranging from 200 to 400 mg/kg, providing neuroprotective and cholesterol-lowering benefits, often exceeding tocopherols in efficacy [5,6] (Table 1). Tocopherols, though present at lower concentrations, synergize with tocotrienols and carotenoids to enhance antioxidant potential [5,6]. Minor bioactive compounds such as phytosterols, squalene, and coenzyme Q10 contribute additional health benefits, including cholesterol-lowering, antioxidant, and skin-protective effects [7] (Table 1). Optimized processing is essential to retain these bioactives, as refining can reduce carotenoid content by up to 70% [3,4].

This quantitative profile underscores the multifunctional bioactivities of RPO, forming the basis for its application in functional foods, nutraceuticals, and cosmetic formulations. Careful selection of processing and storage conditions is essential to maintain its bioactive potential and nutritional quality [12,13] (Table 1).

Although RPO bioactives exhibit promising antioxidant, anti-inflammatory, and lipid-modulating activities, further studies are warranted to evaluate their absorption, distribution, metabolism, excretion, and potential off-target effects (ADMET), particularly for broader pharmaceutical applications.

## 3. Bioactive Properties of RPO

RPO exhibits multiple bioactive properties, mainly due to its carotenoids, tocotrienols, tocopherols, and minor phenolics. These compounds contribute to antioxidant, anti-inflammatory, and antimicrobial activities, which have been well documented in both in vitro and in vivo studies.

### 3.1. Antioxidant Activity

The antioxidant potential of RPO is primarily attributed to carotenoids (alpha-carotene, beta-carotene, lycopene) and tocotrienols. These compounds scavenge reactive ROS, inhibit lipid peroxidation, and stabilize cellular membranes. Quantitative in vitro analysis using DPPH radical scavenging assays showed that crude RPO exhibits 50–70% inhibition at 100 μg/mL, comparable to standard antioxidants such as vitamin C [14,15]. In vivo studies in Wistar rats fed 5% RPO for 12 weeks demonstrated a 35% reduction in hepatic malondialdehyde (MDA) and a 20–30% increase in superoxide dismutase (SOD) and catalase activity compared with control groups [16]. These results indicate that RPO effectively mitigates oxidative stress and enhances endogenous antioxidant defense systems.

### 3.2. Anti-Inflammatory Effects

RPO exhibits anti-inflammatory properties by modulating key signaling pathways. In vitro studies using macrophage and keratinocyte cell lines showed that RPO extracts suppress NF-κB activation, resulting in decreased secretion of proinflammatory cytokines, including TNF-α and IL-6. Dose-dependent inhibition was observed, with 50–100 μg/mL of RPO extract reducing TNF-α secretion by approximately 40% [17]. Tocotrienols additionally downregulate cyclooxygenase-2 (COX-2) expression, further contributing to anti-inflammatory effects [18]. Collectively, these findings support RPO’s potential in managing chronic inflammation and skin disorders. Mechanistic pathways affected by RPO bioactives, including NF-κB, Nrf2, and COX-2, are summarized in Supplementary Table S1.

### 3.3. Antimicrobial Activity

Emerging evidence indicates that RPO exhibits selective antimicrobial activity, particularly against Gram-positive bacteria (Table 2). Minimum inhibitory concentrations (MICs) of RPO were reported as 1.5–3% (*v*/*v*) against *Staphylococcus aureus* and 2–4% (*v*/*v*) against *Listeria monocytogenes* [19,20]. This effect is attributed to the combined action of tocotrienols, carotenoids, and minor phenolic compounds, which can disrupt bacterial membranes and inhibit growth. Furthermore, synergistic interactions with lactic acid bacteria enhance both antimicrobial and probiotic effects, suggesting applications in food preservation, oral care, and skin health products [21].

### 3.4. Mechanistic Insights

These studies collectively demonstrate that RPO is a multifunctional bioactive oil with measurable effects on oxidative stress, inflammation, and microbial inhibition. The major bioactive compounds—carotenoids, tocotrienols, tocopherols, and minor phenolics—act through complementary molecular mechanisms to confer health benefits [14,15,16,17,18,19,20,21]. Through these actions, RPO bioactives reduce oxidative stress, modulate inflammatory responses, support cardiovascular and neuroprotective functions, enhance skin barrier integrity, and protect against microbial pathogens. To provide conceptual clarity, the mechanistic interactions among major RPO bioactives and their targeted molecular pathways are described in this section rather than illustrated as a figure.

Carotenoids (α-carotene, β-carotene, and lycopene) primarily function as antioxidants. They scavenge ROS, inhibit lipid peroxidation, and stabilize cellular membranes, thereby reducing oxidative damage in tissues. Carotenoids also modulate redox-sensitive transcription factors such as NF-κB, leading to decreased expression of proinflammatory cytokines, including TNF-α, IL-6, and IL-1β [14,15,16,17].

Tocotrienols, a potent form of vitamin E, exert anti-inflammatory effects by directly inhibiting NF-κB activation and downregulating cyclooxygenase-2 (COX-2) expression [18]. They also enhance endogenous antioxidant enzymes such as superoxide dismutase (SOD) and catalase, strengthening cellular defense systems against oxidative stress [16,18]. Through these mechanisms, tocotrienols contribute to neuroprotection, cardiovascular health, and attenuation of skin inflammation [22,23,24,25,26,27,28].

Tocopherols, although present at lower concentrations, act synergistically with tocotrienols and carotenoids to stabilize cell membranes and enhance free radical scavenging, amplifying the overall antioxidant and anti-inflammatory capacity of RPO [15,18].

Minor phenolics, squalene, and phytosterols further modulate inflammatory responses and provide cytoprotective effects. These compounds inhibit proinflammatory cytokine production and support skin barrier integrity, hydration, and photoprotection [7,19,20,21,27,28].

Synergistic interactions among these bioactives are critical. The combined presence of carotenoids and tocotrienols enhances ROS scavenging and more effectively suppresses NF-κB-mediated inflammatory signaling than individual compounds. Similarly, phenolics act alongside tocotrienols to regulate cytokine production, reinforcing anti-inflammatory outcomes [17,21].

The major bioactive compounds in RPO not only act through these molecular mechanisms but also exhibit favorable ADMET properties, ensuring effective bioactivity in target tissues. Fat-soluble carotenoids are well absorbed and distributed mainly to the liver and adipose tissue, where they are partially converted to vitamin A [1,2,3]. Tocotrienols and tocopherols show moderate absorption and distribute to the liver, brain, and skin, undergoing hepatic metabolism with low toxicity at dietary levels [4,5,6]. Other bioactives such as squalene, phytosterols, chlorophyll, and polyphenols also display moderate absorption and low toxicity, with excretion primarily via bile or feces [3,5,6,7,8].

Taken together, these pharmacokinetic characteristics complement the mechanistic actions described above, ensuring that the antioxidant, anti-inflammatory, and protective effects of RPO bioactives are effectively realized in vivo. Overall, RPO bioactives act through interconnected pathways that mitigate oxidative stress via ROS scavenging and upregulation of antioxidant enzymes, regulate inflammatory mediators through NF-κB and COX-2 suppression, support cardiovascular and neuroprotective health, maintain skin integrity, and exert antimicrobial activity through membrane disruption and synergistic interactions with beneficial microorganisms [16,17,18,19,20,21,27,28,29].

## 4. Potential Health Applications of RPO

RPO demonstrates promising applications in nutrition, functional foods, nutraceuticals, and cosmetics due to its multifunctional bioactive profile.

### 4.1. Cardiovascular Health

Tocotrienols and carotenoids in RPO contribute to cardiovascular protection by modulating lipid metabolism and oxidative stress. Supplementation with 10–15 mL/day of RPO for 12 weeks in hyperlipidemic patients reduced total cholesterol and low-density lipoprotein (LDL) levels by 15–20%, while increasing high-density lipoprotein (HDL) levels by 5–8% [22,23]. Tocotrienols also inhibit HMG-CoA reductase, the rate-limiting enzyme in cholesterol synthesis, providing a mechanism for lipid-lowering effects [24]. Additionally, antioxidant activity helps prevent LDL oxidation, further reducing atherogenic risk [25].

### 4.2. Neuroprotection

RPO’s tocotrienols have demonstrated neuroprotective properties in both in vitro and in vivo models. Animal studies show that dietary supplementation with RPO (5% *w*/*w*) protects neuronal cells from oxidative damage, reduces ischemia-induced brain injury, and improves cognitive performance in rodent models of Alzheimer’s disease [26,27]. The underlying mechanism involves both antioxidant and anti-inflammatory pathways, including inhibition of ROS and NF-κB signaling [28].

### 4.3. Skin Health and Cosmetic Applications

RPO demonstrates pronounced benefits for skin health and cosmetic applications (Table 3) due to its rich composition of carotenoids, tocotrienols, tocopherols, squalene, and phytosterols. These bioactive constituents exhibit antioxidant, anti-inflammatory, and photoprotective activities that strengthen the skin barrier, enhance hydration, and mitigate visible signs of aging [27,28,29]. A comparative summary of these effects and related health applications is presented in Table 3.

In vitro studies show that extracts of the oil protect keratinocytes and fibroblasts from UV- or chemically induced oxidative stress, lowering intracellular oxidative radicals by 25–40% at concentrations of 50–100 µg/mL [27,28]. Tocotrienols suppress NF-κB activation and downregulate COX-2 expression in epidermal cells, thereby decreasing pro-inflammatory cytokines such as IL-6 and TNF-α that contribute to skin inflammation and photoaging [17,18].

Ex vivo skin models further indicate that topical application enhances barrier integrity, as evidenced by reduced transepidermal water loss (TEWL) and improved hydration—effects likely mediated by squalene and phytosterols that support stratum–corneum lipid organization [27,28]. Clinical studies corroborate these findings: formulations containing 5–10% RPO improve hydration by 15–20%, reduce erythema, and decrease collagen degradation after 4–8 weeks of application [27,28,29]. When co-formulated with complementary antioxidants such as vitamin C or coenzyme Q10, the oil exhibits 25–35% greater radical-scavenging capacity and enhanced photoprotection, highlighting its potential for synergistic cosmeceutical formulations [30,31].

Detailed comparisons of in vitro, in vivo, and clinical evidence for RPO bioactives are summarized in Supplementary Table S1. Collectively, these findings establish RPO as a multifunctional bioactive ingredient suitable for incorporation into anti-aging creams, sunscreens, and hair-care formulations, supporting both protective and reparative skin functions.

These findings collectively underscore the multifunctional health-promoting potential of RPO. By integrating its antioxidant, anti-inflammatory, and lipid-modulating properties, RPO can serve as a valuable dietary component and a bioactive ingredient in functional foods, nutraceuticals, and topical formulations.

### 4.4. Functional Food and Nutraceutical Applications

Due to its multifunctional bioactive properties, RPO is increasingly incorporated into functional foods, beverages, and nutraceuticals. Its stability during moderate heating and resistance to oxidative degradation under proper storage conditions enable its use in cooking oils, fortified spreads, and dietary supplements [14]. Co-formulation with probiotics or other bioactives further enhances health-promoting effects, including immune modulation and antioxidant protection [21].

### 4.5. Considerations for Use

Optimizing processing and storage conditions is crucial to retaining RPO’s bioactive compounds. Unrefined or minimally processed RPO preserves carotenoids and tocotrienols, whereas refining, bleaching, and prolonged high-temperature storage significantly reduce these components [3,4]. Therefore, careful selection of raw materials, extraction methods, and storage practices is essential to maximize its nutritional and functional potential.

### 4.6. Limitations and Challenges

Despite the promising bioactive properties of RPO, several limitations and challenges should be considered:

#### 4.6.1. Variability in RPO Quality Due to Processing

The quality of RPO can vary significantly depending on the extraction and refining processes. Factors such as temperature, refining methods, and storage conditions can affect the concentration and bioavailability of key bioactive compounds like carotenoids and tocotrienols. This variability may influence the consistency and efficacy of RPO in both health and cosmetic applications [3,5,14].

#### 4.6.2. Health and Environmental Concerns

While moderate intake of palm oil within a healthy diet presents no risks for health, concerns persist regarding the environmental impact of palm oil cultivation. Issues such as deforestation, biodiversity loss, and greenhouse gas emissions are associated with palm oil production, particularly in tropical regions. These environmental challenges necessitate sustainable cultivation practices to mitigate adverse effects [32,33].

#### 4.6.3. Conflicting Clinical Evidence

Although in vitro and animal studies have demonstrated the potential health benefits of RPO, human clinical trials are limited and often yield conflicting results. Variations in study design, sample size, and duration contribute to these discrepancies. Further well-designed, large-scale human clinical trials are essential to establish definitive health claims for RPO [22,23].

## 5. Strategies to Optimize Stability and Efficacy of RPO

The bioactive compounds in RPO, including carotenoids, tocotrienols, tocopherols, and polyphenols, are inherently sensitive to environmental factors such as heat, light, and oxygen. Exposure to these factors during processing, storage, and formulation can lead to degradation of carotenoids by 20–50% over 3–6 months at room temperature, significantly reducing antioxidant and therapeutic potential [21,22]. Therefore, maintaining the stability and bioactivity of RPO is crucial for both nutritional and cosmetic applications (Table 4).

Microencapsulation has emerged as a widely applied strategy to protect RPO bioactives from oxidative and thermal degradation. By entrapping RPO within polymeric or carbohydrate-based matrices, microencapsulation can preserve up to 90% of carotenoid content over 12 weeks of storage at ambient conditions [21,22]. Similarly, emulsification techniques, including oil-in-water emulsions and nanoemulsions, enhance the dispersibility and bioavailability of lipophilic compounds, thereby improving absorption and efficacy when incorporated into functional foods or beverages [22,23,30,34].

In cosmetic formulations, the co-formulation of RPO with other antioxidants, such as vitamin C, coenzyme Q10, or polyphenols, has demonstrated synergistic effects. Studies report that combining RPO with vitamin C in topical emulsions increased free radical scavenging capacity by 25–35% compared to RPO alone, while also enhancing skin penetration and photoprotective effects [23,24]. Liposomal incorporation of RPO further stabilizes sensitive bioactives and allows controlled release, improving long-term efficacy in anti-aging and moisturizing products [23,24,31,34].

Several companies utilize RPO in functional foods and cosmetic products. For example, Carotino Group produces RPO-enriched nutritional supplements and personal care ingredients, while Sternchemie GmbH markets its SternRed product line incorporating RPO for both food and topical formulations. Such industrial applications highlight the translational relevance of RPO beyond academic research and demonstrate its feasibility for large-scale production and commercial utilization.

Collectively, these strategies optimize the stability, bioavailability, and efficacy of RPO, ensuring consistent functional performance in dietary and cosmetic products. By selecting appropriate stabilization techniques, it is possible to maintain antioxidant, anti-inflammatory, and antimicrobial efficacy, thereby maximizing the potential of RPO in health-promoting and cosmetic applications. Such approaches improve shelf-life and ensure consistent bioactivity in consumer products.

## 6. Future Directions

Despite the growing body of evidence supporting the health and cosmetic benefits of RPO, several areas warrant further investigation to fully realize its potential.

Standardization of extraction and processing: Optimized techniques are needed to maximize retention of bioactive compounds, as conventional refining and high-temperature processing can result in losses of 20–50% of carotenoids and 15–30% of tocotrienols [30,31]. Future research should explore green extraction methods, such as supercritical CO_2_ and enzymatic-assisted extraction, to enhance yield while preserving nutritional and functional properties. Moreover, the expansion of oil palm plantations can lead to deforestation, increased carbon emissions, and community land conversion [32], highlighting the importance of sourcing RPO sustainably and considering environmental impacts in future research and industrial applications.

Rigorous clinical validation: Preclinical and small-scale human studies indicate cardiovascular, neuroprotective, and dermatological benefits. For example, daily consumption of 15–30 mL of RPO for 8–12 weeks has been associated with reductions in LDL cholesterol by 8–12% and increases in HDL cholesterol by 5–7% [22,23]. Large-scale randomized controlled trials with adequate sample size, dose optimization, and longer duration across diverse populations are essential to validate these effects and strengthen evidence for clinical and nutraceutical applications.

Synergistic formulations: Combining RPO with probiotics, polyphenols, or other nutraceuticals may enhance antioxidant capacity and bioactivity. Early research suggests that pairing RPO with lactic acid bacteria can enhance antimicrobial activity and support gut health, while combination with polyphenols may further increase antioxidant capacity by 20–30% compared to RPO alone [21,30,31]. Future studies should systematically evaluate the bioavailability, stability, and efficacy of such multi-component formulations.

Safety and regulatory considerations: Long-term safety, potential interactions with other dietary components, pharmaceuticals, and cosmetic ingredients, as well as ADMET/dermal sensitivity, must be systematically evaluated. Investigations into sustained delivery systems, such as liposomal and microencapsulated formulations, can improve stability and controlled release of bioactive compounds, enhancing overall efficacy [34]. Regulatory aspects for functional foods, nutraceuticals, and cosmeceuticals should also be addressed to facilitate translation into practical applications.

Summary: Future research should integrate optimized extraction, rigorous clinical trials, synergistic formulation strategies, comprehensive safety assessment, and regulatory considerations. These efforts will consolidate the scientific basis for RPO’s health and cosmetic applications and support its translation into effective functional foods, nutraceuticals, and cosmeceutical products.

## 7. Conclusions

RPO represents a nutritionally and functionally valuable oil distinguished by its richness in carotenoids, tocotrienols, tocopherols, and other minor bioactive constituents. These components collectively confer potent antioxidant activity, as evidenced by in vivo reductions in lipid peroxidation, enhancement of endogenous antioxidant enzymes, and pronounced anti-inflammatory and antimicrobial effects against pathogens such as *Staphylococcus aureus* and *Listeria monocytogenes* [14,15,16,17,18,19,20,21].

Evidence from preclinical and limited clinical studies indicates beneficial impacts on lipid metabolism, cognitive performance, and skin parameters [22,23,24,25,26,27,28,29]. However, further well-designed, large-scale randomized controlled trials are essential to validate these findings across diverse populations. Where data remain limited, interpretations in this review are presented as emerging evidence to reflect the current state of research.

The stability of RPO bioactive compounds remains a key consideration; technological approaches such as microencapsulation, liposomal delivery, and co-formulation with complementary antioxidants have proven effective in maintaining compound integrity and biological efficacy during storage and application [30,31,34].

Taken together, these findings highlight the multifaceted potential of RPO as a valuable ingredient for integration into functional foods, nutraceuticals, and cosmeceutical formulations. Continued interdisciplinary research is warranted to substantiate its therapeutic efficacy and ensure long-term safety in human applications [22,23,24,25,26,27,28,29].

## Figures and Tables

**Table 1:** Typical nutritional composition of RPO.

Component	Typical Range in RPO	Function/Bioactivity	References
Alpha-carotene	150–250 mg/kg	Vitamin A precursor, antioxidant	[1,3,4]
Beta-carotene	200–350 mg/kg	Vitamin A precursor, antioxidant	[2,3,4]
Lycopene	50–100 mg/kg	Antioxidant, cardiovascular protection	[3,4]
Tocotrienols	200–400 mg/kg	Neuroprotective, cholesterol-lowering	[5,6]
Tocopherols	50–100 mg/kg	Antioxidant synergist	[5,6]
Phytosterols	500–1000 mg/kg	Cholesterol-lowering, anti-inflammatory	[7]
Squalene	200–500 mg/kg	Antioxidant, skin-protective	[7]
Coenzyme Q10	10–30 mg/kg	Mitochondrial function, antioxidant	[7]

**Table 2:** Selected bioactive properties of RPO.

Bioactivity	Measurement/Model	Observed Effect/Value	References
Antioxidant	DPPH assay (100 μg/mL)	50–70% radical scavenging	[14,15]
Antioxidant	Wistar rats, 5% RPO, 12 weeks	MDA decreased by 35%; SOD and catalase increased by 20–30%	[16]
Anti-inflammatory	TNF-α secretion in macrophages (50–100 μg/mL)	TNF-α decreased by approximately 40%	[17]
Antimicrobial	MIC against *Staphylococcus aureus*	1.5–3% (*v*/*v*)	[19,20]
Antimicrobial	MIC against *Listeria monocytogenes*	2–4% (*v*/*v*)	[19,20]

**Table 3:** Summary of RPO effects on cardiovascular, cognitive, and skin health.

Health Domain	Model/Measurement	Observed Effect/Value	References
Cardiovascular	Human studies, 8–12 weeks	LDL-C decreased by 10–15%; HDL-C increased by 5–8%	[9,15,16]
Cardiovascular	HMG-CoA reductase inhibition	Inhibition of cholesterol biosynthesis	[16]
Cognitive/Neuroprotective	Animal model, 2–5% dietary RPO	Cognitive performance increased; neuronal oxidative damage decreased by 20–25%	[17,18]
Skin Health/Cosmetic	Topical RPO 5–10%	Hydration increased by 15–20%; barrier function increased	[27,28,29]
Skin Health/Cosmetic	UV protection, anti-aging	Erythema decreased; collagen degradation decreased	[27,28,29]

**Table 4:** Strategies for enhancing stability and efficacy of RPO.

Strategy	Mechanism/Description	Observed Outcome	References
Microencapsulation	Encapsulation in polymeric or carbohydrate matrix	Carotenoids preserved at approximately 90% over 12 weeks	[21,22]
Emulsification/Nanoemulsion	Formation of oil-in-water or nanoemulsions	Improved bioavailability and dispersibility	[22,23]
Antioxidant Co-formulation	Combining with vitamin C, coenzyme Q10, polyphenols	Free radical scavenging increased by 25–35%; skin penetration enhanced	[23,24]
Liposomal incorporation	Encapsulation in phospholipid bilayers	Controlled release; improved photoprotection	[23,24]

## Data Availability

No new data were created or analyzed in this study. Data sharing is not applicable to this article.

## Supplementary Materials

The following supporting information can be downloaded at: https://www.mdpi.com/article/10.3390/molecules30224402/s1, Table S1: Mechanistic and evidence overview of Red Palm Oil (RPO) bioactives; Table S2: ADMET properties of major bioactive compounds in red palm oil.
