# Supplementary material for: Red Palm Oil: Nutritional Composition, Bioactive Properties, and Potential Applications in Health and Cosmetics: A Narrative Review
Source: Molecules. 2025 Nov 14;30(22):4402. doi: 10.3390/molecules30224402 (PMC12655212; doi:10.3390/molecules30224402)
Supplement: Supplementary file 1 [file molecules-30-04402-s001.zip › molecules-3943817-supplementary.pdf]

Supplementary Table S1 – Mechanistic and evidence overview of Red Palm Oil (RPO) bioactives.

Bioactive Component	Mechanism / Pathway	Observed Effect	Evidence Type	References
Carotenoids (α-, β-carotene, lycopene)	ROS scavenging; Nrf2 activation; β-carotene metabolism	↑ Endogenous antioxidant enzymes; ↓ Oxidative stress	In vitro, in vivo, limited human	[1,2,3,8,28,34]
Tocotrienols (Vitamin E)	NF-κB inhibition; COX-2 suppression	↓ Pro-inflammatory cytokines (TNF-α, IL-6); ↓ Inflammation; neuroprotection	In vitro, in vivo, human	[1,3,9,10,17,18,25,26,29]
Tocopherols (Vitamin E)	Lipid peroxidation inhibition	↓ Cellular oxidative damage; ↑ antioxidant defense	In vitro, in vivo	[1,3,7,14,24]
Minor phenolics, squalene, phytosterols	Synergistic antioxidant & anti-inflammatory effects	↑ Skin barrier, ↑ Hydration, photoprotection	In vitro, in vivo	[3,4,6,7]
Combined RPO bioactives	Modulation of lipid metabolism; ADMET effects	↓ LDL, ↑ HDL; cognitive protection; immune modulation	In vivo, limited human	[1,5,16,22,23]
Cosmetic / skin-related outcomes	Barrier repair, hydration, anti-aging	↑ TEWL protection, ↑ Moisture retention, ↓ Wrinkle formation	In vitro, limited clinical	[6,27,34]
Quality & Processing Variability	Degradation during storage/refining	Changes in bioactive content; variable antioxidant potential	Analytical / review	[3,5,12,13,14,30,31,34]
Limitations / Environmental Concerns	Sustainability, deforestation, social impacts	Conflicting evidence; environmental issues	Review / observational	[32,33]
Controversial / Conflicting Health Evidence	Cardiometabolic outcomes	Mixed findings on cardiovascular risk	Review / human studies	[5,22]

Supplementary Table S2. ADMET properties of major bioactive compounds in red palm oil.

Bioactive Compound	Absorption	Distribution	Metabolism	Excretion	Toxicity	Reference
Alpha-carotene	Good, fat-soluble, enhanced by dietary lipids	Liver, adipose tissue	Converted to vitamin A in intestine/liver	Bile/feces	Low	[1,2,3]
Beta-carotene	Good, fat-soluble	Liver, adipose tissue	Converted to vitamin A	Bile/feces	Low	[1,2,3]
Alpha-tocotrienol	Moderate	Liver, brain, skin	Hepatic metabolism	Bile/feces	Generally safe at dietary levels	[4,5,6]
Gamma-tocotrienol	Moderate	Liver, brain, skin	Hepatic metabolism	Bile/feces	Generally safe	[4,5,6]
Tocopherols	Moderate	Liver, plasma	Hepatic metabolism	Bile/feces	Safe at dietary levels	[4,5,6]
Squalene	Moderate	Liver, skin	Minimal metabolism	Urine/feces	Low	[7,8]
Phytosterols	Low-moderate	Plasma, liver	Minimal metabolism	Feces	Low	[7,8]
Chlorophyll	Low	Liver, kidney	Partial degradation in gut	Feces	Low	[3]
Polyphenols	Moderate	Plasma	Hepatic metabolism	Urine/feces	Low	[5,6]
